# Imaging Flow Cytometry as a Quick and Effective Identification Technique of Pollen Grains from *Betulaceae*, *Oleaceae*, *Urticaceae* and *Asteraceae*

**DOI:** 10.3390/cells11040598

**Published:** 2022-02-09

**Authors:** Iwona Gierlicka, Idalia Kasprzyk, Maciej Wnuk

**Affiliations:** 1 Department of Biology, Institute of Biology and Biotechnology, College of Natural Sciences, University of Rzeszow, Pigonia 1, 35-310 Rzeszow, Poland; igierlicka@ur.edu.pl (I.G.); ikasprzyk@ur.edu.pl (I.K.); 2 Department of Biotechnology, Institute of Biology and Biotechnology, College of Natural Sciences, University of Rzeszow, Pigonia 1, 35-310 Rzeszow, Poland

**Keywords:** imaging flow cytometry, aerobiology, pollen grains, allergenic pollen

## Abstract

Despite the continuous and intensive development of laboratory techniques, a light microscope is still the most common tool used in pollen grains differentiation. However, microscopy is time-consuming and needs well-educated and experienced researchers. Other currently used techniques can be categorised as images and non-images analysis, but each has certain limitations. We propose a new approach to differentiate pollen grains using the Imaging Flow Cytometry (IFC) technique. It allows for high-throughput fluorescence data recording, which, in contrast to the standard FC, also enables real-time control of the results thanks to the possibility of digital image recording of cells flowing through the measuring capillary. The developed method allows us to determine the characteristics of the pollen grains population based on the obtained fluorescence data, using various combinations of parameters available in the IDEAS software, which can be analysed on different fluorescence channels. On this basis, we distinguished pollen grains both between and within different genera belonging to the *Betulaceae*, *Oleaceae*, *Urticaceae* and *Asteraceae* families. Thereby, we prove that the proposed methodology is sufficient for accurate, fast, and cost-effective identification and potentially can be used in the routine analysis of allergenic pollen grains.

## 1. Introduction

Aerobiology is an interdisciplinary branch of science covering issues related to the movement of biotic elements and particles of organic origin in the atmosphere, including primarily the study of their source, dispersal, and impact on living organisms and the environment [1,2]. Pollen is an important component of aeroplankton responsible for inhalant allergies. For this reason, monitoring its concentration in the atmosphere is especially meaningful in allergology because it allows, above all, the development of pollen calendars and forecast of pollen concentrations, which can be useful for the prevention and treatment of allergies. Furthermore, aerobiological research is widely used in other areas of science, including biogeography, (paleo)botany, (paleo)climatology, ecology, phenology, and in practice such as agriculture, horticulture, melissopalynology, forestry, forensic and bioterrorism [1].

Usually, attempts to identify pollen grains take into account their morphology, including size, shape, and surface sculpture, and also type, number, and arrangement of apertures, which can be observed using light microscopy, still invariably considered as “the gold standard” of analysis [3,4]. However, these methods have many shortcomings. 

Most of all, analyses are time-consuming and require well-educated and experienced scientists. Furthermore, they do not allow one to distinguish many pollen grains at the species level and are often identified only to taxonomic genus or even family [5,6].

For many years, intensive work has been carried out on developing a tool enabling automatic identification and counting of pollen grains. Many of the tools are based on image processing of microscope images combined with machine learning [7,8]. Recently, few European monitoring stations implemented automatic and remote working Rapid E aerosol sensors. Its technology is based on a scattered light pattern and deep UV laser-induced fluorescence [9]. It should be emphasised that the weakness of the automatic systems is the inability to reanalyse pollen samples [7]. Here some literature about automated microscopic analyses, e.g., with the HUND real-time pollen monitoring, is missing. For the HUND system, it is possible to reanalyse samples at a later time point [10,11].

**Figure 1:**
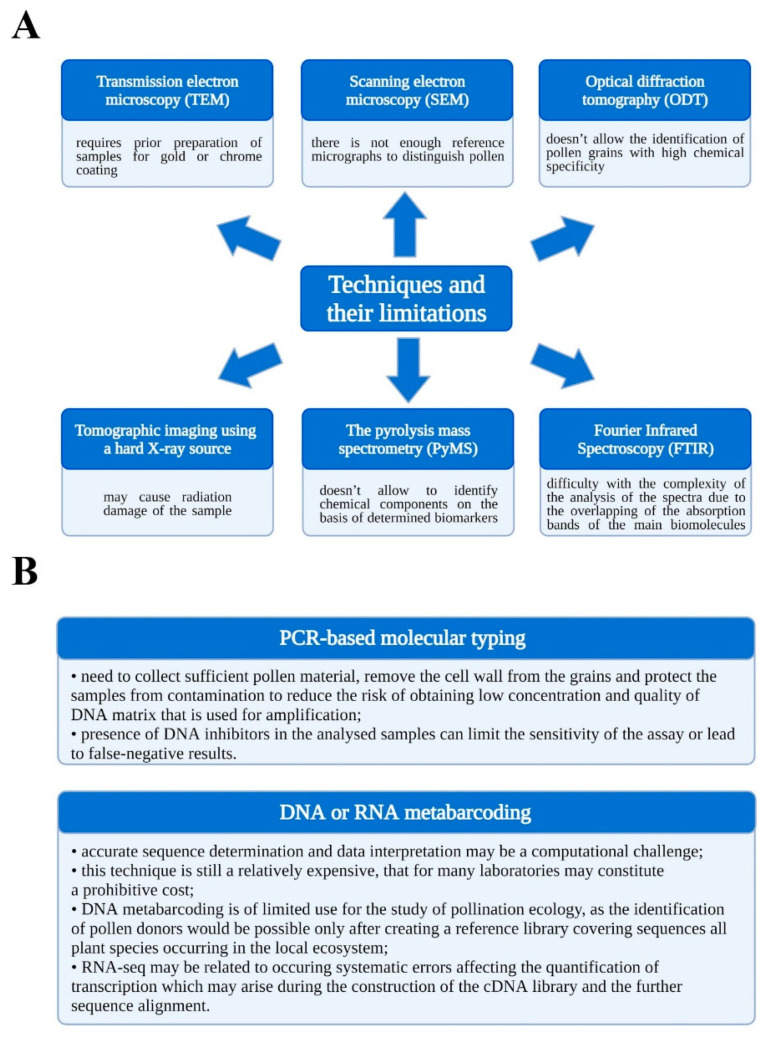
Techniques of pollen grains identification and their limitations (**A**), Limitations of molecular techniques for pollen grains identification (**B**).

Other advanced methods, which are listed in Figure 1A, also have some limitations, as well as require highly specialised and expensive equipment [12,13,14,15,16].

The identification of pollen grains is also possible using molecular detection techniques which are usually carried out using PCR-based molecular typing [17,18]. Modern sequencing technologies and bioinformatic tools made DNA metabarcoding capable of identifying pollen grains with high throughput even at the species level [19,20]. In turn, RNA metabarcoding (RNA-seq) allows differentiating pollen donors based on genotyping, which uses sequencing data. Molecular markers such as SNPs used for this purpose can even distinguish mutants and specific cultivars alleles [21,22,23]. However, applying metabarcoding as a ritual identification test is also subject to certain limitations (Figure 1B) [17,18,23,24,25]. Recently, the presence of small RNAs such as microRNA (miRNA) in pollen grains of various plant species has been discovered, and their sequencing has been attempted [26,27,28]. Perhaps, in the future, their sequencing data will also constitute a convenient material for pollen grain differentiation.

An alternative to the above-mentioned methods seems to be flow cytometry (FC). FC is based on the principle of hydrodynamic focusing, in which the cells suspended in the shielding fluid concentrate under the influence of a vacuum in the centre of the stream and flow laminar into the measuring chamber. There, they are irradiated with a focused laser beam, which results in the phenomenon of light scattering and excitation of cell-related fluorochromes. The light signal is measured by appropriate detectors, converted into electrical signals, amplified, and analysed using special software [29,30,31,32]. FC has already been used successfully to distinguish grains on the basis of DNA analysis [33,34,35]. However, traditional flow cytometry does not have a well-validated real-time control system of the origin of the fluorescent signal because the results are based solely on the parameters received from the device without the possibility of optical verification of the analysed objects [24]. In addition, this technique cannot distinguish plant species, but it has been noticed that it gains this ability after combining with microscopy [35,36].

Imaging flow cytometry (IFC), as a modification of FC, offers many more application possibilities. IFC enriches traditional FC with high-resolution morphological information, providing a hybrid tool that allows single-cell fluorescent and morphological analysis, and can be easily applied in many scientific fields [37,38]. The ability to pass up even to 5000 particles per second through the measuring capillary, coupled with the possibility of analysing up to 10–15 spectral bands and additional scatter signals, can enable quick and efficient analysis of multiple parameters [39,40]. Moreover, although this method for cell detection usually requires the use of dyes intercalating with their nucleic acids, it is not necessary for pollen grain analysis because they can generate autofluorescence themselves. This ability to fluorescence is mainly the result of laser beam excitation of sporoderm-related fluorophores such as carotenoid pigments, phenolic compounds, chlorophyll, and proteins. For this reason, there is no risk of chemical alteration or disruption of the internal pigments of the sample by the dye [12,41].

Given the applicability of imaging flow cytometry in aerobiology, we investigated the possibility of distinguishing the allergenic pollen chosen using this technique. This approach allowed us to evaluate whether such a methodological solution would be effective for quick identification of pollen grains among different botanical families, as well as closely related species.

## 2. Materials and Methods

The tree pollen of the *Betulaceae* (*Alnus incana*, *Alnus glutinosa*, *Corylus avellana*, *Betula pubescens*, *Betula pendula*, *Betula utilis*, *Carpinus betulus*) and *Oleaceae* (*Fraxinus excelsior*) families, as well as the pollen of herbaceous plants of the *Asteraceae* (*Taraxacum officinale*, *Solidago* sp., *Artemisia* sp., *Ambrosia artemisiifolia*) and *Urticaceae* (*Urtica dioica*) families were considered. The analysis centred on strong allergenic pollen (*Betula* sp., *Artemisia* sp., *Ambrosia* sp.), which present cross-reactivity with other allergens and are similar in their morphology [5]. The pollen grains collection was preceded by botanist identification of each donor plant. The pollen grains from flowers were collected directly into the tube. To validate the IFC results, all pollen samples were examined using standard microscopy analysis according to the procedure described in [5]. All pollen samples were identified with and without fuchsin staining for additional control of the quality of microscopic analysis [5].

The IFC has been performed according to the following protocol. Pollen samples (<1 mg) were suspended in 1 mL of 70% ethanol (frozen at −20 °C), mixed using a vortex for 60 s and allowed to fix for 40 min at room temperature. After fixation, the samples were centrifuged for 4 min at 13,000 rpm, ethanol was drained, and 200–500 μL of PBS solution was added to them and vortexed for 30 s. The samples prepared in this way were examined using an Amnis^®^ FlowSight^®^ imaging flow cytometer with manual measurement, and the acquired data were analysed using IDEAS software version 6.2.187.0 (Luminex Corporation, Austin, TX, USA). To reveal the pollen grains fluorescence profiles, a blue laser with a wavelength of 488 nm and a power of 30 mW and a few bandpass fluorescence filters were used as follows: 457/45 nm (Channel 1), 528/65 nm (Channel 2), 577/35 nm (Channel 3), 610/30 nm (Chanel 4), 702/85 nm (Channel 5), and 762/35 nm (Channel 6). Additionally, a red laser with a wavelength of 642 nm and a power of 30 mW and two bandpass fluorescence filters were used 702/85 nm (Chanel 11) and 762/35 nm (Chanel 12). Moreover, a 785 nm laser was used for the side scatter channel (SSC). The speed of a single analysis was ranged from 50 objects/s to 500 objects/s.

**Figure 2:**
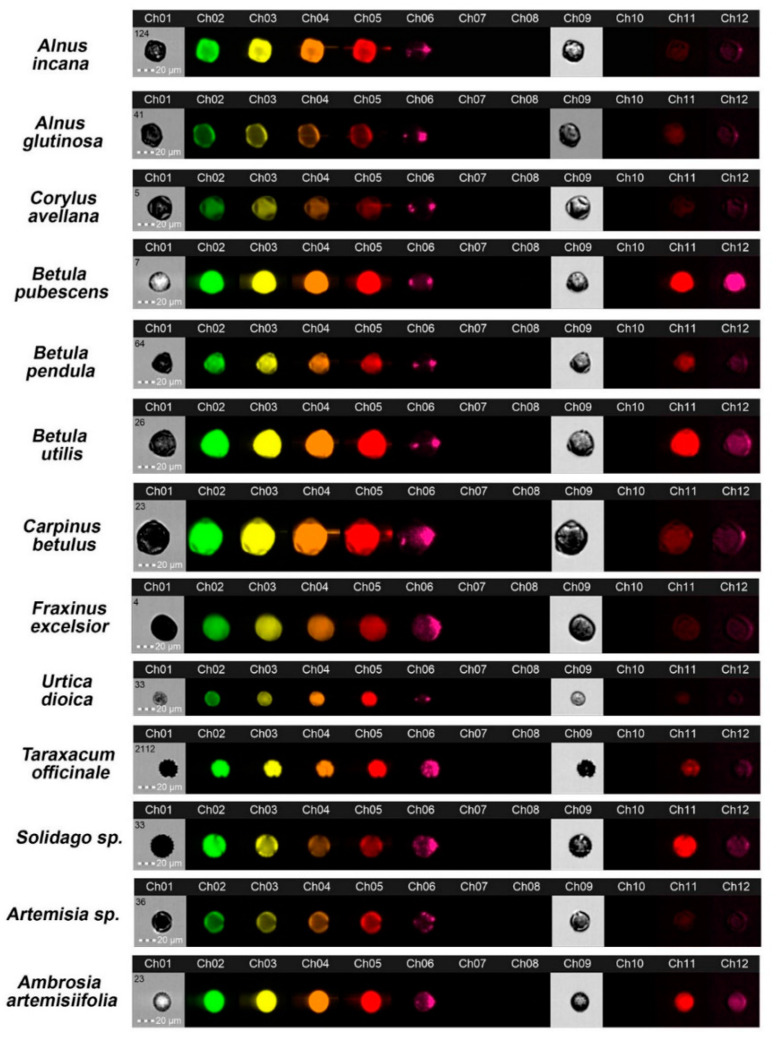
Images of pollen grains examined in individual fluorescent channels taken at ×20 magnification with the imaging flow cytometer.

Pollen grains profiles were acquired for 2000 events without gating using INSPIRE software. Then, the raw data saved as “Raw Image Files” were analysed using IDEAS software. The selection of the R1 focused population was carried out based on the fluorescence intensity in channel 2 (Figure 2). The detailed analysis strategy was provided in the Supplementary Information (Supplementary File ‘.ast’).

## 3. Results and Discussion

The plant species and/or genera were identified based on botanical analyses. The collected pollen grains were subjected to routine microscopic examination under a light microscope (Supplementary Figures S1 and S2). Microscopic analyses identified specific morphological characteristics, including size, shape, the presence of apertures and a slightly visible surface sculpture (Figures S1 and S2). Furthermore, it was shown that some morphological characteristics of pollen grains can be visualised better after staining the preparations with a fuchsin staining solution (Supplementary Figure S2). The characteristics of pollen features of the tested plants were provided as in legend in Supplementary Figure S2. The analyses showed that the part of the pollen grains are very difficult to differentiate with the conventional microscopic method. Additionally, the analysis of, e.g., 2000 pollen grains on a single preparation takes an average of 3–5 h and requires prior experience. A serious problem during microscopic analysis is the different orientations on the vertical and horizontal axis of pollen grains the overlap, and adherence of the pollen grains to each other, which may mask certain features and, consequently, makes analysis and accurate counting difficult. In practice, it is hardly possible to identify pollen grains to the species level, but only the genus or even family. It is undoubtedly a cheap technique but requires a good quality microscope and camera. The quality of the analyses depends very much on the researcher’s experience.

Due to some limitations of standard techniques used, there is a need for new solutions to identify pollen grains in a simple, cheap, and more time-effective manner. An essential task is to enable the automation of the analysis as much as possible to reduce the risk of human error. Taking into account the above, the IFC method seems to be a very good solution, which, unlike FC, has a good real-time control system of the analysed results, thanks to the possibility of digital image recording of cells flowing through the measuring capillary [37,38]. IFC was previously widely used in science for analysis in the biology of human cells [42], yeast [43], algae [44] and bacteria [24]. Moreover, recently this technique, in combination with the deep learning algorithm, was successfully applied in the identification of pollen grains belonging to families: *Lamiaceae*, *Orobanchaceae*, *Rubiaceae*, *Gentianaceae*, *Asteraceae*, *Campanulaceae*, *Apiaceae*, *Dipsaceae*, *Primulaceae*, *Caryophyllaceae*, *Fabaceae*, *Rosaceae*, *Violaceae*, *Clusiaceae*, *Parnassiaceae*, *Cistaceae* and *Ranunculaceae* [6]. The authors claimed 96% accuracy of pollen grain identification, including those that are impossible or difficult distinguished using microscopy. We proposed a new identification solution carried out based on the obtained fluorescence data, using various combinations of parameters available in the IDEAS software, such as: ‘Aspect ratio vs. Area’, ‘Aspect ratio Intensity vs. Area’, ‘Normalised Frequency vs. Intensity’, ‘Intensity vs. Intensity’, which can be analysed on different fluorescence channels.

**Figure 3:**
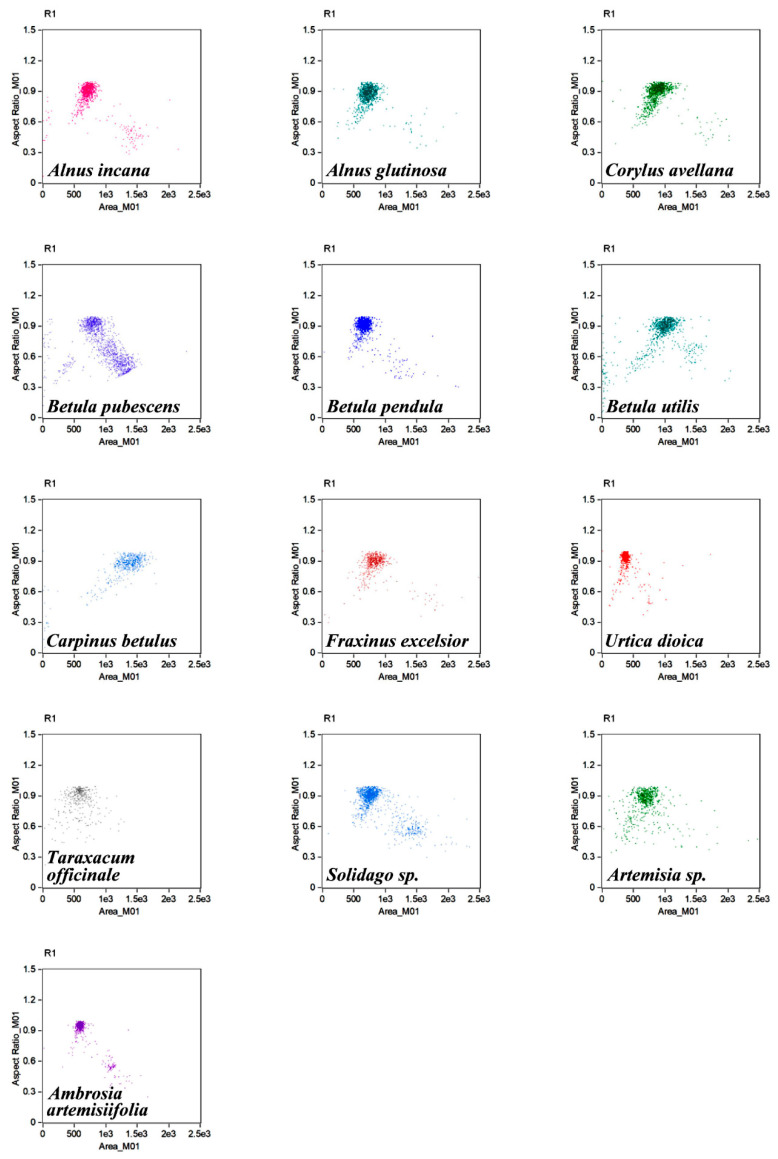
Characterisation of pollen grain populations of species studied using IFC. The best-focused objects (R1) were gated according to the parameter ‘aspect ratio’ (channel 1, BF, *y*-axis) versus the ‘area’ parameter (channel 1, BF, *x*-axis).

The Amnis^®^ FlowSight^®^ apparatus with a CCD camera allowed real-time registration of single pollen grain images on individual fluorescent channels; examples are shown in Figure 2. Cell populations were characterised, and the best-focused objects (R1) were gated according to the parameter ‘aspect ratio’ (channel 1, BF, *y*-axis) versus the parameter ‘area’ (channel 1, BF, *x*-axis) (Figure 3).

**Figure 4:**
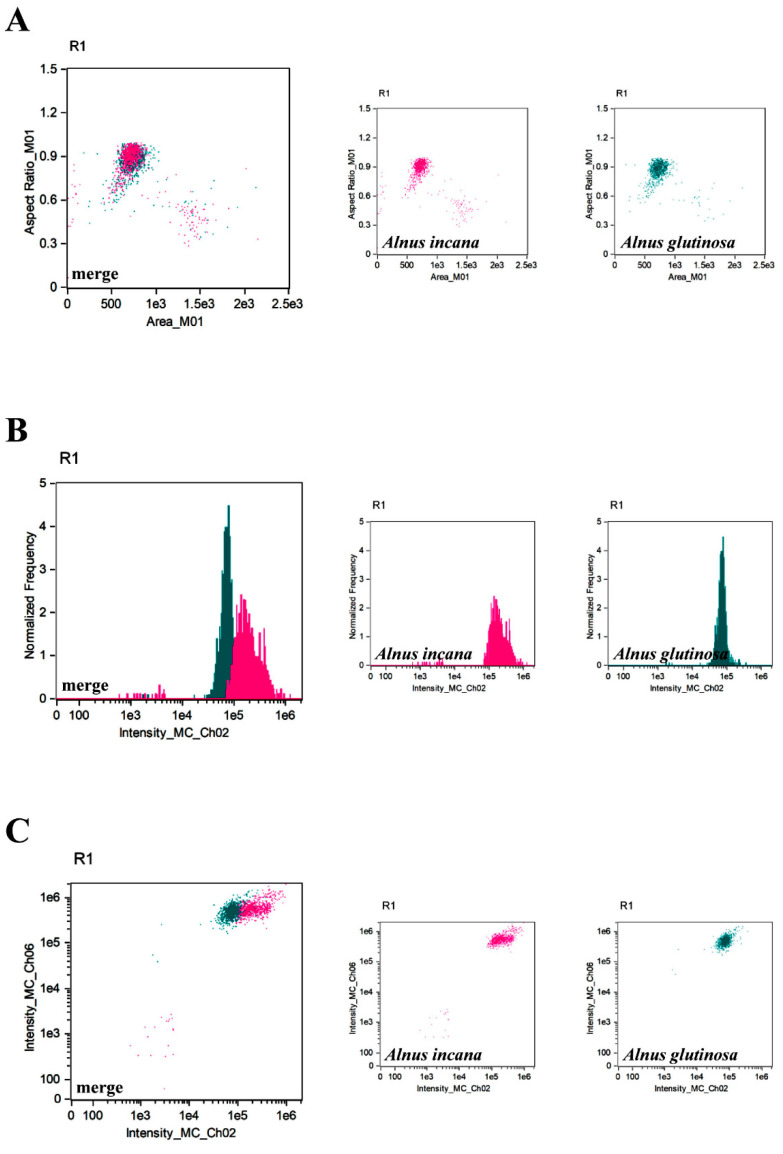
Characterisation of pollen grains populations of two species of alder: *Alnus incana* (pink) and *Alnus glutinosa* (teal) using IFC. Three types of analysis (different parameters) were considered, namely ‘aspect ratio’ parameter in channel 1 (BF, *y*-axis) versus ‘area’ parameter (channel 1, BF, *x*-axis) (**A**), ‘normalized frequency’ parameter (channel 1, BF, *y*-axis) versus fluorescence ‘intensity’ parameter in channel 2 (*x*-axis) (**B**) and the fluorescence ‘intensity’ parameter (channel 6, SSC, *y*-axis) versus fluorescence ‘intensity’ parameter (channel 2, *x*-axis) (**C**). Separate tree profiles and merged profiles are shown.

**Figure 5:**
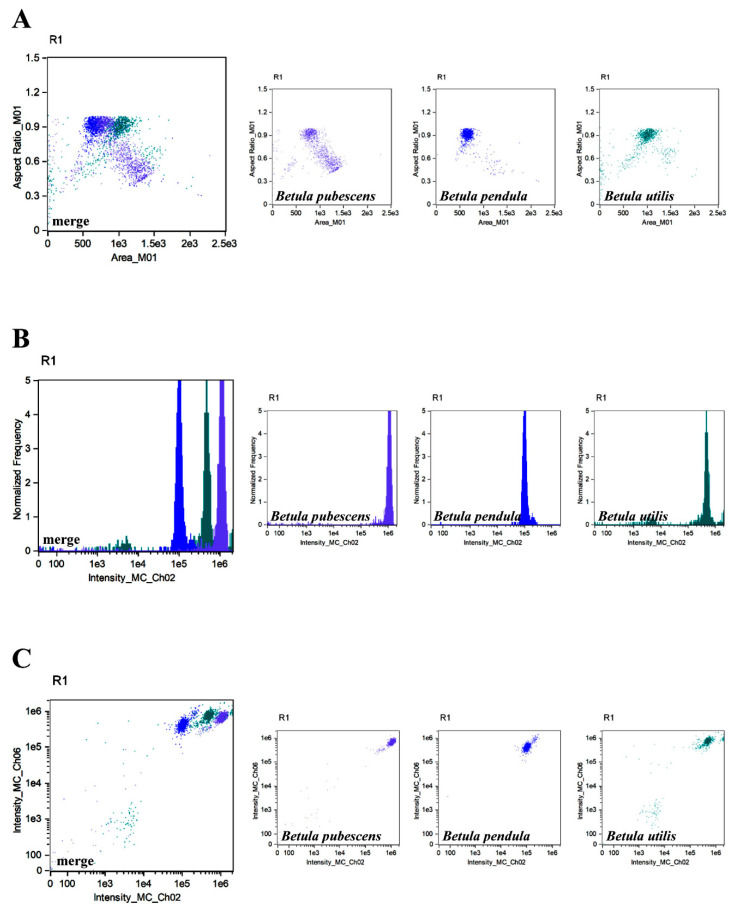
Characterisation of pollen grains populations of three species of birch using IFC. Three types of analysis (different parameters) were considered, namely the parameter ‘aspect ratio’ in channel 1 (BF, *y*-axis) versus the parameter ‘area’ (channel 1, BF, axis *x*) (**A**), ‘normalized frequency’ parameter (channel 1, BF, *y*-axis) versus fluorescence ‘intensity’ parameter in channel 2 (*x*-axis) (**B**) and the ‘intensity’ fluorescence parameter (channel 6, SSC, *y*-axis) versus fluorescence ‘intensity’ parameter (channel 2, *x*-axis) (**C**). *Betula pubescens* (violet), *Betula pendula* (navy blue), and *Betula utilis* (teal) separate profiles and merged profiles are shown. Colocalisation analysis allows differentiation of birch at the species level.

**Figure 6:**
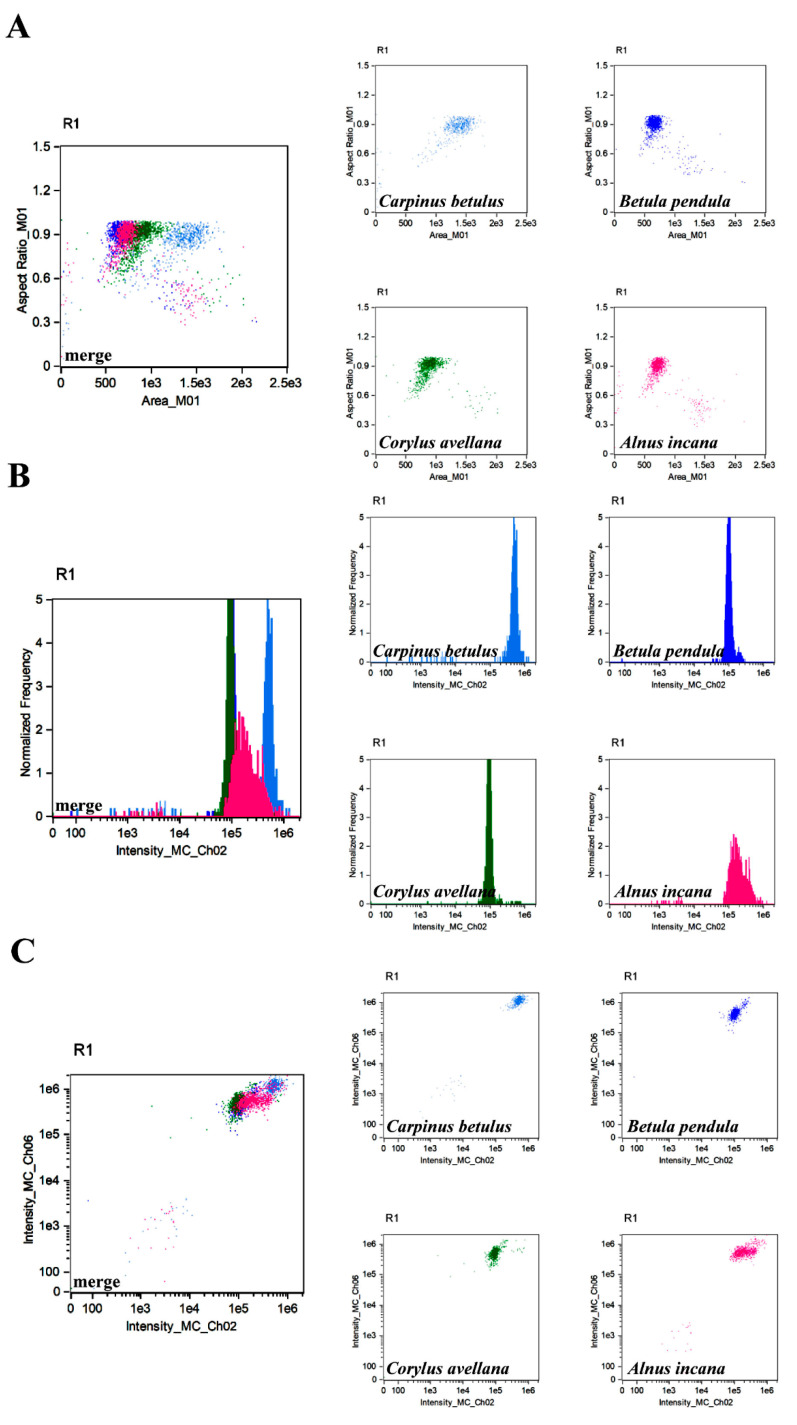
Colocalisation analysis of four species of trees belonging to the *Betulaceae* family using IFC. Three types of analysis (different parameters) were considered, namely the parameter ‘aspect ratio’ in channel 1 (BF, *y*-axis) versus ‘area’ parameter (channel 1, BF, *x*-axis) (**A**), ‘normalized frequency’ parameter (channel 1, BF, *y*-axis) versus fluorescence ‘intensity’ parameter in channel 2 (*x*-axis) (**B**) and the ‘intensity’ fluorescence parameter (channel 6, SSC, *y*-axis) versus fluorescence ‘intensity’ parameter (channel 2, *x*-axis) (**C**). *Carpinus betulus* (blue), *Betula pendula* (navy blue), *Corylus avellana* (green), and *Alnus incana* (pink) separate profiles and merged profiles are shown.

**Figure 7:**
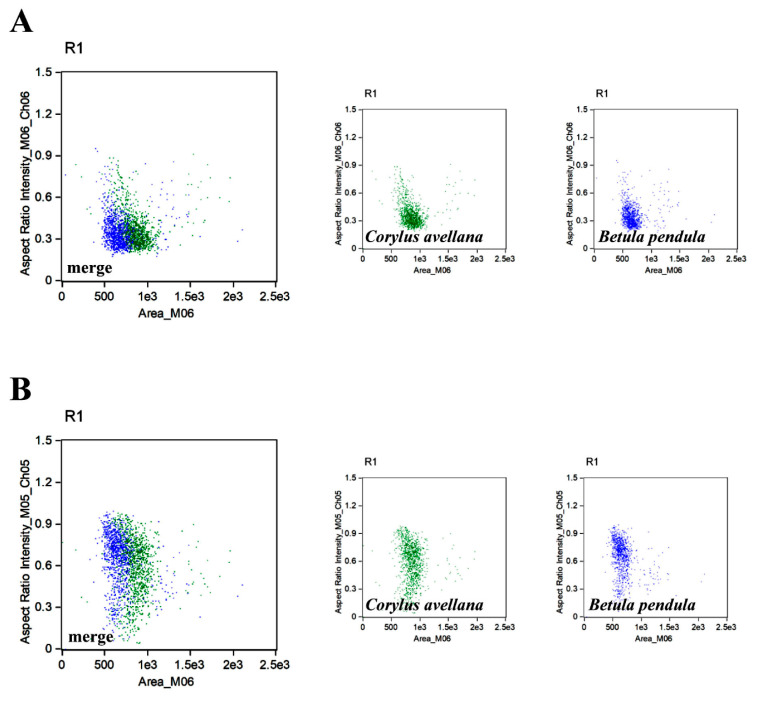
Colocalisation analysis *Corylus avellana* population (green) with *Betula pendula* population (navy blue) using IFC. Due to the close colocalisation of these species (Figure 6), additional parameters were considered, namely the ‘aspect ratio intensity’ parameter in channel 6 (SSC, *y*-axis) versus the ‘area’ parameter (channel 6, SSC, *x*-axis) (**A**) and the ‘aspect ratio intensity’ parameter in channel 5 (*y*-axis) versus the ‘area’ parameter (channel 6, SSC, *x*-axis) (**B**). Separate and merged profiles are shown.

To evaluate pollen grain profiles and to distinguish between different species, the following parameters were considered: parameter ‘aspect ratio’ in channel 1 (BF, *y*-axis) versus parameter ‘area’ (channel 1, BF, *x*-axis), parameter ‘normalised frequency’ (channel 1, BF, *y*-axis) versus the ‘intensity’ fluorescence parameter in channel 2 (*x*-axis) and the ‘intensity’ fluorescence parameter (channel 6, SSC, *y*-axis) versus the ‘intensity’ fluorescence parameter (channel 2, *x*-axis). These analysis options were saved as a template (Supplementary File ‘.ast’). The use of these three combinations of parameters proved to be sufficient to accurately differentiate species in most of the analysed cases because after placing individual profiles of pollen grains on one graph, their populations were distinguishably located and did not overlap with each other; thus, they were considered the standard parameters’ setup. Most of all, this approach allowed to differentiate pollen grains at the species level, e.g., *Alnus* species: *A. incana* and *A. glutinosa* (Figure 4) and *Betula* species: *B. pubescens*, *B. pendula*, and *B. utilis* (Figure 5). This was almost impossible by microscopy, although the pollen grains of some species of birch differ in size [45]. Moreover, it enabled us to distinguish pollen grains of more species belonging to *Betulaceae* such as *A. incana* and *C. betulus* from *B. pendula* and *C. avellana*, respectively (Figure 6). All are cross-reacting due to the chemical similarity of their allergens, and in the temperate climate zone their pollen seasons partially overlap [46]. For this reason, quick and accurate pollen identification is especially desired in pollen concentration forecasting. The pollen grains of the *B. pendula* and *C. avellana* trees are trizonoporate and easy to distinguish under a light microscope [5]. Due to the overlapping IFC profiles, it was quite difficult to distinguish them. In this case, we decided to use additional parameters, such as: ‘aspect ratio intensity’ parameter in channel 6 (SSC, *y*-axis) versus ‘area’ parameter (channel 6, SSC, *x*-axis) (Figure 7A) and ‘aspect ratio’ intensity parameter in channel 5 (*y*-axis) versus ‘area’ parameter (channel 6, SSC, *x*-axis) (Figure 7B). It allowed for a more accurate separation of the profiles and their simple distinction. Thus, in the case of species that are difficult to distinguish using the selected standard combination of parameters, other of the many parameters available in the IDEAS software can be used, which greatly expands the analysis possibilities.

**Figure 8:**
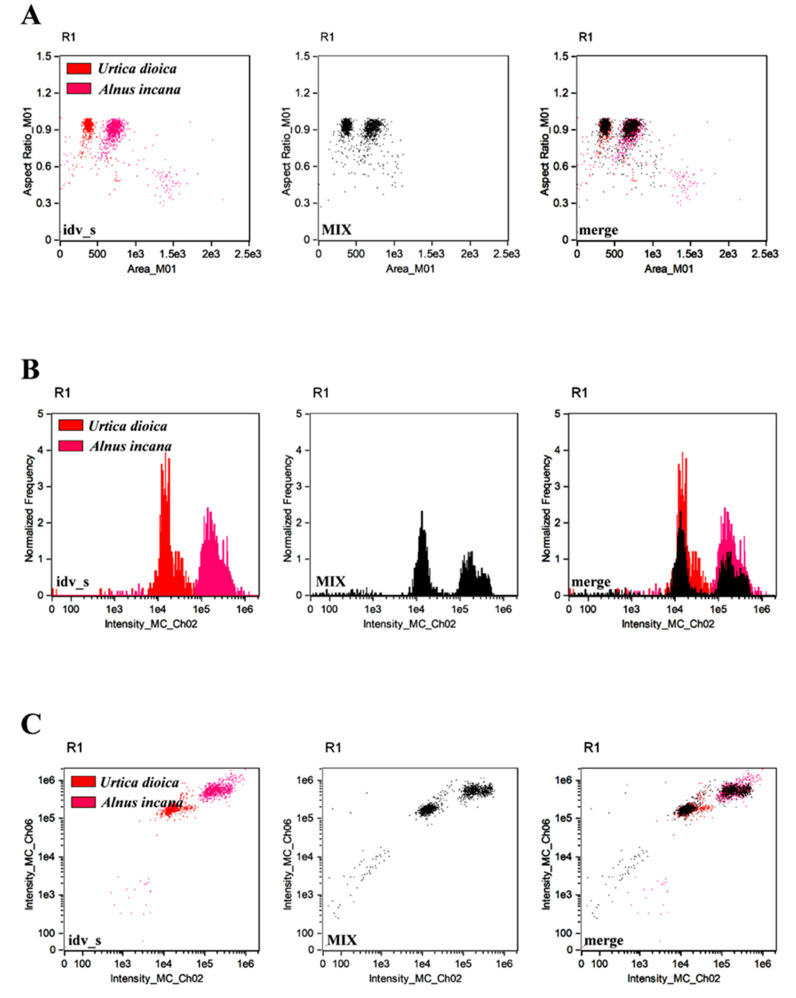
Analysis of the profiles of *Alnus incana* and *Urtica dioica*. Pollen grains were generated based on their individual samples (idv_s) and their prepared mixture (MIX). Three types of analysis (different parameters) were considered, namely the parameter ‘aspect ratio’ in channel 1 (BF, *y*-axis) versus ‘area’ parameter (channel 1, BF, *x*-axis) (**A**), ‘normalized frequency’ parameter (channel 1, BF, *y*-axis) versus fluorescence ‘intensity’ parameter in channel 2 (*x*-axis) (**B**) and the ‘intensity’ fluorescence parameter (channel 6, SSC, *y*-axis) versus fluorescence ‘intensity’ parameter (channel 2, *x*-axis) (**C**). The third graph on the left shows the overlapping profiles that are consistent with each other, which confirms the usefulness of the proposed IFC-based assay.

Due to the high potential for identifying pollen grains based on individual profiles merged in a separate collocation, we decided to conduct an analysis verifying the effectiveness of differentiation after passing a pollen mixture of various species through the IFC apparatus. For this purpose, we chose pollen of different botanical families: *A. incana* and *U. dioica*, both zonoporate but differ, among others, in exine thickness, number of pores, and size [5]. Firstly, their profiles were generated on the basis of their individual samples (idv_s) and their prepared mixture (MIX) with the use of previously specified standard setup of parameters, and after that, we merged created plots (Figure 8). This procedure revealed overlapping profiles that were consistent with each other, which confirms the usefulness of the proposed IFC-based assay.

The taken above, the main advantage of this IFC is the reduction in the time required to perform analyses compared to microscopy techniques. In addition, the IFC analysis is analysed automatically in the same way that software, regardless of the type of sample and researcher’s experience, allows us to maintain the objectivity of the analyses. The reliability of the method is higher than that of microscopy analysis. The individual profile of pollen grains can be deposited in an international database repository and can be freely available for everyone in the future. In this way, IFC can allow us to reduce the impact of human error. The measurements performed enable the creation of individual profiles of each type of pollen grains based on numerous discriminating parameters. In IFC analyses, various fluorescence parameters can be used to analyse pollen grains. IFC also can visually verify photos of pollen grains similar to those used in conventional microscopy techniques. The program generates numerous data that are easy to verify, control, and compare by various laboratories. IFC enables the use of machine learning algorithms to refine the analysis of new and/or hard-to-distinguish pollen. Only basic skills in distinguishing pollen grains and reading charts are required for working with IFC. Moreover, the preparation protocol requires the use of non-hazardous reagents (only ethanol and PBS), and it does not require additional staining compared to the microscopic method (initial sample preparation requires the use of chemical reagents, which are hazardous for people) in which staining is often used (the preparation of stained glycerogelatin requires the use of phenol or azide and fuchsine which are potentially carcinogenic). In addition, the ethanol fixation step in the IFC can even be omitted without causing any disturbance in the subsequent analysis. IFC enables percentage estimation by determining the number of grains of a specific species in relation to the very large amount of all analysed grains. Pollen grains for analysis may be kept long term in the freezer. Although some researchers indicate that the fluorescence of pollen grains can vary over time due to their ageing, which could be a potential obstacle to the IFC method; however, our research did not reveal any variation in IFC profiles after comparing some species that differed in age by several years (data was not presented in the article). Additionally, it is worth mentioning that from the perspective of the economic aspect of purchasing equipment, the cost of the IFC apparatus and high-resolution optical microscope, although quite high, is relatively comparable. In turn, from the view of the maintenance aspect associated with hiring and training of staff, the cost is much higher when using the microscopic method for routine analysis, which is mainly due to the high amount of manual work and the time-consuming nature of the analyses.

## 4. Conclusions

In conclusion, the pollen grain profiles analysis based on the IFC using the proposed selected parameters can be successfully used to differentiate the pollen grains in an easy, fast, reliable and cost-effective manner. This confirms that the presented method may be implemented in routine aerobiological analysis. The fast analysis and the accuracy of identification are advantages especially useful in preparing reliable reports on the concentration of allergenic pollen for physicians and their patients.

## Data Availability

The data are available within the Supplementary data files or from the corresponding authors upon reasonable request. Supplementary File ‘.ast’.

## Supplementary Materials

The following supporting information can be downloaded at: https://www.mdpi.com/article/10.3390/cells11040598/s1, Supplementary File S1 ‘.ast’: Analysis template. Supplementary File S2: Morphological characteristic pollen grains using the light microscope method.
