# Supplementary material for: Imaging Flow Cytometry as a Quick and Effective Identification Technique of Pollen Grains from Betulaceae, Oleaceae, Urticaceae and Asteraceae
Source: Cells. 2022 Feb 9;11(4):598. doi: 10.3390/cells11040598 (PMC8870286; doi:10.3390/cells11040598)
Supplement: Supplementary file 1 [file cells-11-00598-s001.zip › cells-1578824-supplementary_file 2.pdf]

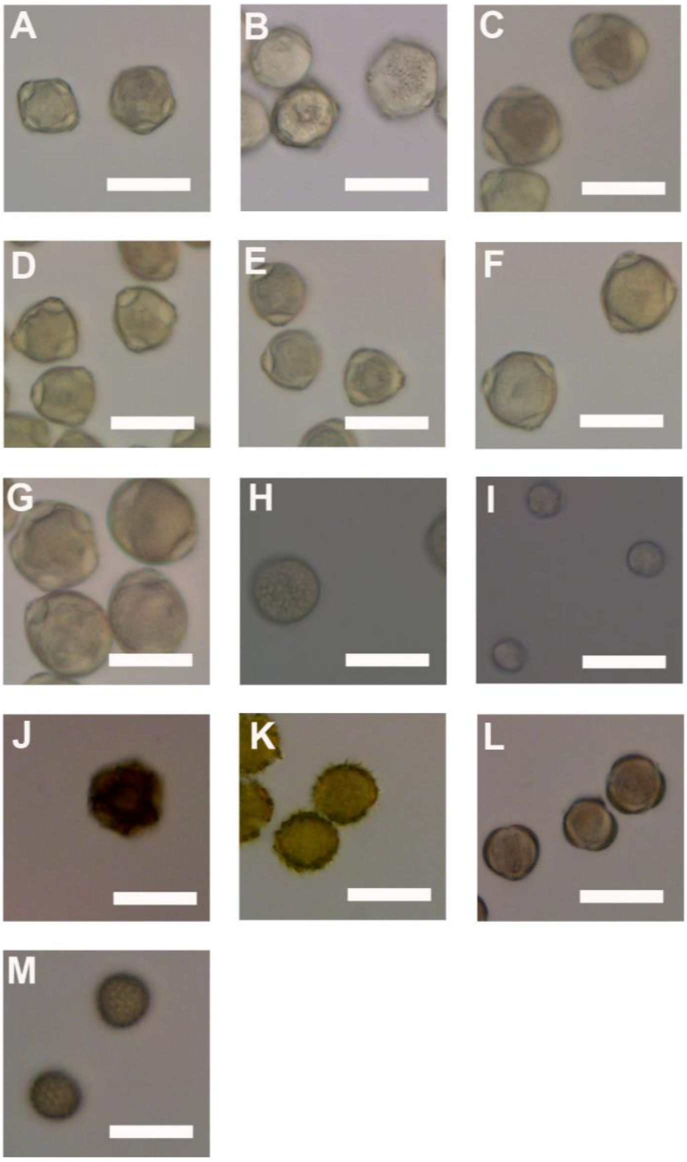

Figure S1. The pollen grains without staining examined in light microscope Olympus BX61 under 40x objective. A) *Alnus incana;* B) *Alnus glutinosa;* C) *Corylus avellana;* D) *Betula pubescens; E) Betula pendula;* F) *Betula utilis;* G) *Carpinus betulus; H) Fraxinus excelsior; I) Urtica dioica; J) Taraxacum officinale; K) Solidago* sp.; L) *Artemisia* sp.; M) *Ambrosia artemisiifolia.* Scale bar = 30µm

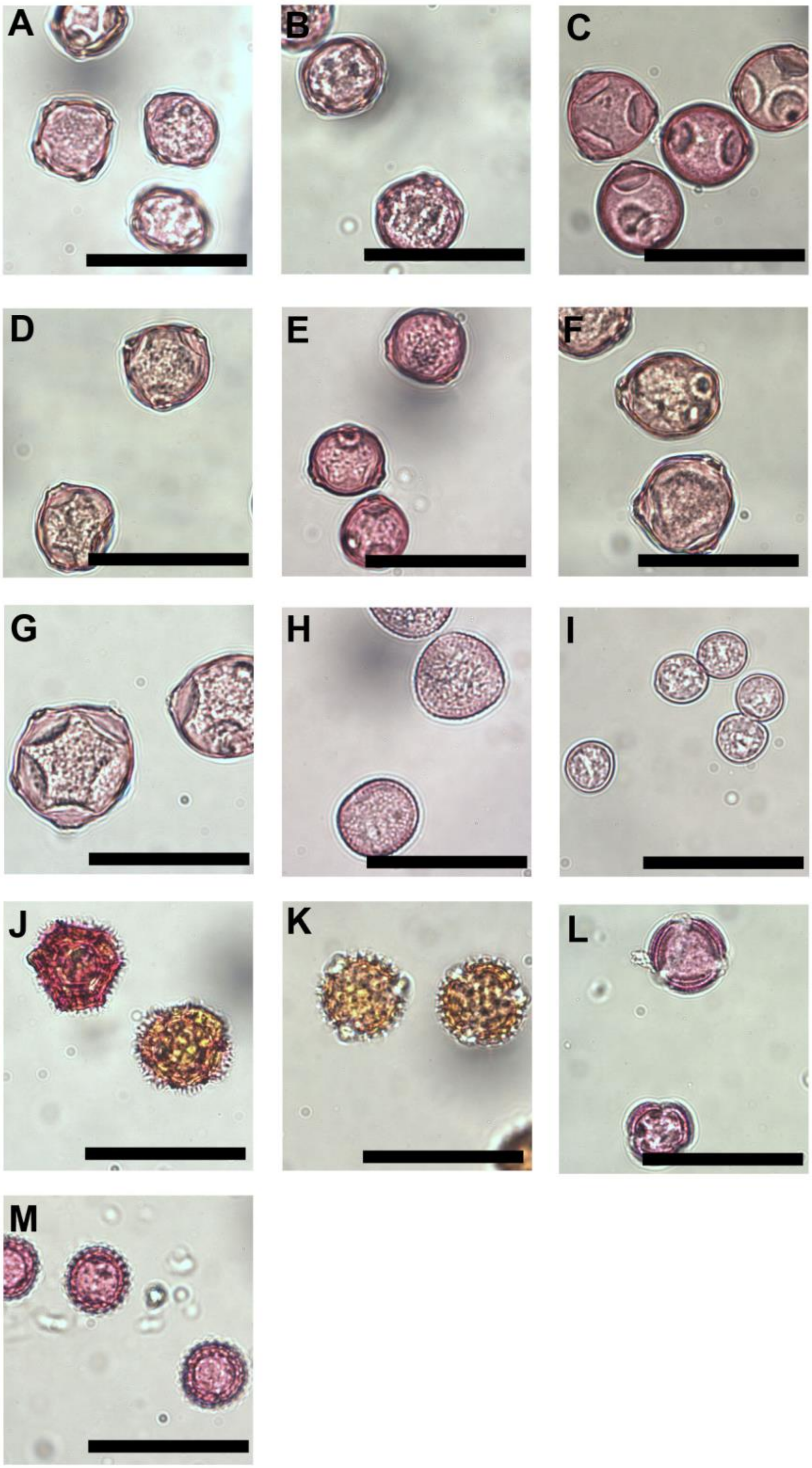

Figure S2. The pollen grains after staining with fuchsin examined in light microscope Nikon Eclipse 80i under 40x objective. A) *Alnus incana;* B) *Alnus glutinosa;* C) *Corylus avellana;* D) *Betula pubescens; E) Betula pendula;* F) *Betula utilis;* G) *Carpinus betulus; H) Fraxinus excelsior; I) Urtica dioica; J) Taraxacum officinale; K) Solidago* sp.; L) *Artemisia* sp.; M) *Ambrosia artemisiifolia.* Scale bar = 50µm

According to (Frenguelli, G., & Kasprzyk, I. (2015). Description of pollen grains. In I. Kasprzyk & M. Smith (Eds.). *Manual for aerobiology*. Wyd. Univ. Rzeszow):

- ***Alnus sp.*** – (tetra) penta -zonoporate, isopolar, oblate or oblate-spheroidal, small (21-30 µm); exine is thin, protruding aspidate pores, the pores are vestibulate; sexine forms thickened streaks (arci) swinging pores;
- ***Corylus sp.*** – tri-zonoporate, isopolar, suboblate, in polar view subtriagular, small (20-30 µm); exine is thin, thickened around pores and forms an aspis;
- ***Betula sp.*** – tri-zonoporate, isopolar, suboblate, small (on average 18-26 µm); exine is thin; the pores are circular, surrounded by an aspis; the exine forms the vestibulum near porus;
- ***Carpinus sp.*** – tetra (penta)-zonoporate, isopolar, suboblate, medium sized (30-36 µm); exine is rather thin, thickened around the pores to form an aspis; pores are circular with operculm;
- ***Fraxinus sp***. – tri (tetra)-zonocolpate, isopolar, suboblate, small (18-25 µm); exine is reticulate, meshes varying in size, but not diminishing toward furrows; furrowns are narrow;
- ***Urtica sp.*** – tri (tetra)-zonoporate, isopolar, oblate spheroidal, small (12-16 µm); exine is very thin; pores are circular, small;
- ***Taraxacum sp.*** – tri(tetra)-zonoporate, isopolar, oblate-spheroidal, small (18-30 µm); depressed areas surrounded by thick exine ridges; pores are circular;
- ***Solidago sp.*** – tri-zonocolporate, isopolar, spheroidal, small (15-25 µm); exine is medium thickness with finely pointed spines; furrows narrowing outside the pore; pores are circular;
- ***Artemisia sp***. – tri-zonocolporate, isopolar, spheroidal or oblate spheroidal, small (17-20 µm); exine is thick with spines, thinning toward the furrows; furrows are narrowing outside the pore; pores are located one in the middle of each furrow;
- ***Ambrosia sp.*** – trizonocolporate, isopolar, oblate spheroidal, small (18-21 µm); exine with very short blunt spines; furrow is short to very short, narrow; pores are located one in the middle of each furrows.
